# Impact of SARS-CoV-2 vaccination of children ages 5–11 years on COVID-19 disease burden and resilience to new variants in the United States, November 2021–March 2022: a multi-model study

**DOI:** 10.1016/j.lana.2022.100398

**Published:** 2022-11-22

**Authors:** Rebecca K. Borchering, Luke C. Mullany, Emily Howerton, Matteo Chinazzi, Claire P. Smith, Michelle Qin, Nicholas G. Reich, Lucie Contamin, John Levander, Jessica Kerr, J. Espino, Harry Hochheiser, Kaitlin Lovett, Matt Kinsey, Kate Tallaksen, Shelby Wilson, Lauren Shin, Joseph C. Lemaitre, Juan Dent Hulse, Joshua Kaminsky, Elizabeth C. Lee, Alison L. Hill, Jessica T. Davis, Kunpeng Mu, Xinyue Xiong, Ana Pastore y Piontti, Alessandro Vespignani, Ajitesh Srivastava, Przemyslaw Porebski, Srini Venkatramanan, Aniruddha Adiga, Bryan Lewis, Brian Klahn, Joseph Outten, Benjamin Hurt, Jiangzhuo Chen, Henning Mortveit, Amanda Wilson, Madhav Marathe, Stefan Hoops, Parantapa Bhattacharya, Dustin Machi, Shi Chen, Rajib Paul, Daniel Janies, Jean-Claude Thill, Marta Galanti, Teresa Yamana, Sen Pei, Jeffrey Shaman, Guido España, Sean Cavany, Sean Moore, Alex Perkins, Jessica M. Healy, Rachel B. Slayton, Michael A. Johansson, Matthew Biggerstaff, Katriona Shea, Shaun A. Truelove, Michael C. Runge, Cécile Viboud, Justin Lessler

**Affiliations:** a The Pennsylvania State University, University Park, PA, USA; b Johns Hopkins University Applied Physics Laboratories Laurel, MD, USA; c Northeastern University, Boston, MA, USA; d Johns Hopkins University, Baltimore, MD, USA; e Harvard University, Cambridge, MA, USA; f University of Massachusetts Amherst, Amherst, MA, USA; g University of Pittsburgh, Pittsburgh, PA, USA; h École polytechnique fédérale de Lausanne, Lausanne, Switzerland; i University of Southern California, Los Angeles, CA, USA; j University of Virginia, Charlottesville, VA, USA; k University of North Carolina at Charlotte, Charlotte, NC, USA; l Columbia University, New York, NY, USA; m University of Notre Dame, Notre Dame, IN, USA; n CDC COVID-19 Response Team, Centers for Disease Control and Prevention, Atlanta, GA, USA; o U.S. Geological Survey, Laurel, MD, USA; p Fogarty International Center, National Institutes of Health, Bethesda, MD, USA; q University of North Carolina at Chapel Hill, Chapel Hill, NC, USA

**Keywords:** SARS-CoV-2, COVID-19, Vaccination, Variant emergence, Scenario projection, Modeling

## Abstract

**Background:**

The COVID-19 Scenario Modeling Hub convened nine modeling teams to project the impact of expanding SARS-CoV-2 vaccination to children aged 5–11 years on COVID-19 burden and resilience against variant strains.

**Methods:**

Teams contributed state- and national-level weekly projections of cases, hospitalizations, and deaths in the United States from September 12, 2021 to March 12, 2022. Four scenarios covered all combinations of 1) vaccination (or not) of children aged 5–11 years (starting November 1, 2021), and 2) emergence (or not) of a variant more transmissible than the Delta variant (emerging November 15, 2021). Individual team projections were linearly pooled. The effect of childhood vaccination on overall and age-specific outcomes was estimated using meta-analyses.

**Findings:**

Assuming that a new variant would not emerge, all-age COVID-19 outcomes were projected to decrease nationally through mid-March 2022. In this setting, vaccination of children 5–11 years old was associated with reductions in projections for all-age cumulative cases (7.2%, mean incidence ratio [IR] 0.928, 95% confidence interval [CI] 0.880–0.977), hospitalizations (8.7%, mean IR 0.913, 95% CI 0.834–0.992), and deaths (9.2%, mean IR 0.908, 95% CI 0.797–1.020) compared with scenarios without childhood vaccination. Vaccine benefits increased for scenarios including a hypothesized more transmissible variant, assuming similar vaccine effectiveness. Projected relative reductions in cumulative outcomes were larger for children than for the entire population. State-level variation was observed.

**Interpretation:**

Given the scenario assumptions (defined before the emergence of Omicron), expanding vaccination to children 5–11 years old would provide measurable direct benefits, as well as indirect benefits to the all-age U.S. population, including resilience to more transmissible variants.

**Funding:**

Various (see acknowledgments).

**Research in context**

**Evidence before this study**

In late 2021, policy makers in the United States were considering an expansion of age-eligibility for SARS-CoV-2 vaccination to include children aged 5–11 years, but estimates of the population-level benefits of such a policy change did not exist. As of March 27, 2022, our search of PubMed using keywords “SARS-CoV-2” AND “vaccine effectiveness” AND “children 5 to 11” yielded 37 articles. Many of these articles report safety and efficacy results for clinical trials in other age groups. Seven articles refer directly to the 5–11-year age group. Three studies comment on or report recommendations, one reports an observational study on hospitalization, and one reports vaccine efficacy. Two modeling studies considered indirect effects of childhood vaccination, yet one was set in Australia and the other focused on school community-level mitigation strategies. Further, these two studies each considered a single model and did not account for heterogeneity in epidemic trajectories or vaccination uptake rates across the U.S.

**Added value of this study**

The COVID-19 Scenario Modeling Hub convenes multiple modeling teams to project long-term (6 months or greater) trajectories of the pandemic at national and state levels under various scenarios. In September 2021, nine teams generated projections under the same set of scenario assumptions of the population-level impacts of vaccinating children aged 5–11 years (starting in November 2021) with and without potential emergence of a more transmissible variant. Projections for cases, hospitalizations, and deaths in the U.S. between September 2021 and March 2022 across scenarios with and without vaccination in children aged 5–11 years were compared to estimate the reductions in disease burden associated with expanded vaccine eligibility, with and without variant emergence. Models projected measurable reductions in national cases, hospitalizations, and deaths in scenarios with an expanded vaccination program (range of point estimates across clinical outcomes, 7.2–12.3%). Disease burden reductions projected by models were more pronounced in the presence of a more transmissible variant and in younger age groups.

**Implications of all the available evidence**

Scenario projection results indicate that expanding vaccine eligibility to children aged 5–11 years can provide substantial direct and indirect benefit at the population level. During the projection period, the benefits of high coverage in this age group are likely to be greater in the presence of new variants or during periods of high population transmission; actual realized benefits will depend both on the extent of vaccine coverage and effectiveness, and characteristics of the circulating variants.

## Introduction

SARS-CoV-2 vaccines have contributed to reducing serious outcomes of COVID-19, including severe disease, hospitalization, and death in the United States.^1^ COVID-19 vaccination started in late December 2020, and demand largely surpassed supply through the early months of 2021.^2^ Groups at higher risk, including health care workers and individuals aged ≥65 years, were prioritized to receive SARS-CoV-2 vaccines first. To further direct the first vaccine doses to those most at risk, on December 20, 2020, the Centers for Disease Control and Prevention (CDC), Advisory Committee on Immunization Practices [ACIP] recommended that COVID-19 vaccines initially be offered to persons aged ≥75 years and non–health care frontline essential workers.^3^ Vaccine uptake, particularly in persons aged ≥75 years, increased quickly, contributing to a shift in the age distribution of severe cases by April 2021.^4^^,^^5^ Vaccine emergency-use authorization was expanded by the U.S. Food and Drug Administration (FDA) to include persons 16 years of age or older in April 2021, and persons aged 12–15 years on May 10, 2021.^6^

Despite encouraging signs of a receding pandemic in the U.S. in spring and early summer 2021, emergence of the Delta variant led to renewed COVID-19 risk overall, and particularly among children <12 years old, for whom the vaccine was not yet available. For example, COVID-19 hospitalizations per 100,000 in the U.S. increased among children and adolescents from 0.3 in June to 1.4 in late August 2021^7^; these surges were likely driven by increased transmissibility and severity of the Delta variant.^8^^,^^9^ Thus, despite relatively high coverage in the previously eligible population (approximately 65% of eligible individuals in the U.S. receiving two doses of primary series [Pfizer and Moderna] or one dose of Janssen vaccines by September 2021^2^), there was reason to believe that the expansion of vaccination to children 5–11 years old could have an appreciable impact on the U.S. epidemic. However, the magnitude of this impact remained unclear given overall build-up of acquired immunity, potential differences in age-specific transmission, and the size of this group (7.6% of the U.S. population). Children 5–11 years old began receiving the Pfizer-BioNTech COVID-19 vaccine in the U.S. immediately following the corresponding recommendation from the Advisory Committee of Immunization Practices made on November 2, 2021.^10^ Even several months after initiation of the 5–11 year old vaccination program, assessments of potential national benefits of this policy remain limited. A recent modeling study investigated the impact of childhood vaccination in Australia, a unique setting due to low immunity from prior infection.^11^ Another modeling study assessed the impact of vaccination on school interventions but did not explore national- or state-level benefits.^12^ A single-model study evaluated impacts of school-aged children vaccination in the U.K.^13^ Several other modeling studies have considered vaccination effects in the global context.^14^^,^^15^

In anticipation of authorization and recommendations for expanding vaccination to children 5–11 years old in the U.S. (e.g., FDA and CDC)^10^ members of the COVID-19 Scenario Modeling Hub undertook a multiple-model approach to assess potential effects of immunizing children 5–11 years old and generated information for federal authorities (prior to authorization on October 29, 2021^10^). Our approach is particularly well-suited to generating projections in periods of high uncertainty in disease epidemiology and behavior, by considering multiple epidemiologic scenarios and aggregating results over multiple models. While it has been more than a year since vaccination of children 5–11 years old began in the U.S., substantial value remains in documenting this multiple-model effort and corresponding synthesis of results.

## Methods

### Overview of scenario hub and epidemiological assumptions

The COVID-19 Scenario Modeling Hub (http://covid19scenariomodelinghub.org) was established in December 2020 to generate 6-month-ahead projections of the COVID-19 trajectory in the U.S. under scenarios capturing different epidemiological and intervention assumptions.^16^ For each round of scenarios, multiple modeling groups are convened in an open call to produce projections of weekly cases, hospitalizations, and deaths at both the state- and national-levels. The round discussed in this paper (the ninth round) focused on childhood vaccination, with team-specific projections made by nine modeling teams (see Supplement for complete list of team names) for the period September 12, 2021–March 12, 2022. Round 9 considered four scenarios, including scenarios with and without vaccination in children 5–11 years old with administration beginning on November 1, 2021 in the U.S., and with and without the emergence of a hypothetical more transmissible variant in the U.S. on November 15, 2021.^17^ The hypothetical variant was assumed to be 50% more transmissible than viruses circulating at the start of the projection period (see Table 1 for additional scenario details).

**Table 1:** Specification of the four scenarios included in the COVID-19 Scenario Modeling Hub's Round 9 projections.

	The same mix of variants circulate throughout the projection period. No change in virus transmissibility.	A more transmissible variant emerges, comprising 1% of circulating viruses on **Nov 15, 2021**. The new variant is **1.5X** as transmissible as viruses circulating at the beginning of the projection period.
Vaccination among 5–11 year-olds is approved and immunization begins on Nov 1, 2021. Each state's uptake rate reflects the percent coverage increases observed for 12–17 year-olds since distribution began on May 13, 2021.	A	C
No vaccination for children under 12 years.	B	D

The four scenarios (A, B, C, and D) differed in whether or not they included vaccination of children 5–11 years old, and whether or not the same mix of variants circulated throughout the projection period, September 2021–March 2022 or a new, more transmissible variant emerged.

In scenarios with vaccination of children 5–11 years old, uptake rates in children were specified to reflect those reported by the CDC for 12-17 year-olds at the state level since administration began for that age group on May 13, 2021^2^ (i.e., assumed to match the state-specific uptake curve in older children offset to begin November 1, 2021). Projected uptake in individuals over 12 years was at each team's discretion, but informed by Pulse and CovidCast hesitancy surveys.^18^^,^^19^ State-level vaccination coverage for older individuals was implemented to reflect reports from the CDC.^2^ Age-group specific resolution depended on each team's modeling assumptions, but vaccine saturation levels were assumed to be consistent with observed data. Booster vaccines were not included in order to focus on the potential effects of vaccination in children 5–11 years old.

Delta-variant specific vaccine effectiveness (VE) against infection, symptoms, and severe outcomes also were at team discretion for all age groups (see Supplemental Table S1); although estimates based on U.S. and U.K. studies were provided for guidance.^20^, ^21^, ^22^, ^23^ In the absence of VE estimates for children when scenarios were developed, VE in children 5–11 years old was assumed to match those of older age groups. Vaccine effectiveness estimates were also assumed to remain the same for the hypothetical new variant as for those assumed for the Delta variant. Additional assumptions were at the discretion of teams, based on their best scientific judgment, so that uncertainty in these areas would be reflected in the projections. This included assumptions about seasonal effects, including school terms, non-pharmaceutical intervention usage, and waning immunity.

For example, most teams assumed no reactive interventions or behaviors, but two teams incorporated a probability of self-isolation for symptomatic individuals. Similarly, most teams did not model non-pharmaceutical interventions specific to young age groups explicitly. A variety of methods were used to incorporate school-aged children contact rates including age-specific contact matrices and temporal changes in contact rates due to school terms and holiday closures in the U.S. Notable model-specific variability was present in the characterization of transmissibility, seasonality, and case ascertainment parameters. Further, the age structure of each model varied; most models had two to three age classes representing children under 18 years old; while two models had only one age class and another two models had 18. Additional details on team-specific assumptions are provided in meta-data available in the data repository associated with this study^17^ (also see Supplemental File S1 and Supplemental Table S2). Data through September 11, 2021 were used for model calibration.

### Ensemble estimates

**Fig. 1:**
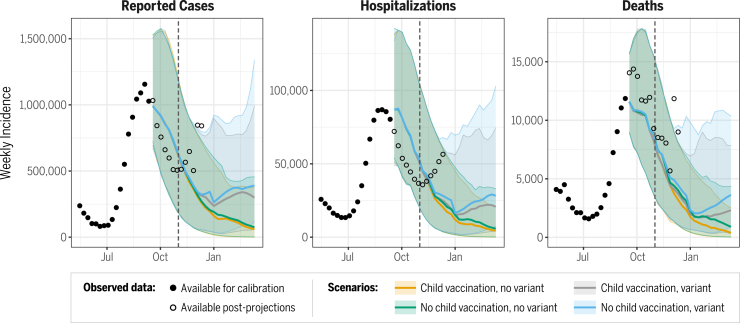
Projected cases, hospitalizations, and deaths for all ages at the national-level (September 12, 2021–March 12, 2022). Median ensemble projections for each scenario displayed as lines, 95% projection intervals displayed as shaded regions. Observed ground truth data available for model calibration (solid points) and after model fitting (open circles) through December 11, 2021 (after which Omicron became prevalent in the U.S., departing from specified scenarios). The start date for vaccination of children 5–11 years old, November 1, 2021, is indicated by a dashed line. See Fig. S1 for ensemble median and 50% projection intervals separated by scenario and the Round 9 tab of the COVID-19 SMH website^16^ for additional visualization functionality. Ensemble median, 50% and 95% credible intervals are provided for cumulative cases, hospitalizations, and deaths over the projection period in Supplemental Fig. S3.

Probabilistic projections (see Supplemental Methods) were reported by each of the nine modeling teams for each scenario, outcome metric, location, and week over the 26-week projection period (September 12, 2021–March 12, 2022). Ensemble projections were generated using a trimmed linear opinion pool method, where cumulative probabilities are averaged at a given value across all of the distributions submitted by the modeling groups, with the highest and lowest probabilities excluded before averaging.^24^^,^^25^ Weekly ensemble distributions were used to create time series projections (Fig. 1) for incident and cumulative outcomes (Fig. S3).

### Evaluation of the benefits of vaccine expansion to children 5–11 years old

To summarize the projected overall benefits of the vaccine program expansion across all nine modeling teams, we used a standard meta-analytic approach with random effects.^26^ Briefly, for each model and location (nationally and for all 50 states individually) we estimated the mean difference in cumulative incidence and the mean incidence ratio between scenarios with and without children 5–11 years old vaccinated, stratified by presence or absence of the new variant, for the portion of the projection period following the assumed start date of childhood vaccination (i.e., from November 1, 2021). Model-specific variance for the mean difference was estimated as the sum of the variances at each timepoint (conservatively assuming zero covariance), while variance of the incidence ratio was estimated using the delta method^27^; both methods were scaled to standard errors using the number of replicates. Model-specific estimates and standard errors were combined via random effects meta-analysis using restricted maximum likelihood (REML); the same procedures were followed for best approximating the direct effect of vaccine expansion (within the younger age group that most closely matched the 5-11-year-old population, e.g., 0-11 year-olds, 5-11 year-olds, projections submitted by five teams). Full details of the methodological approach are found in the Supplementary methods section.

All projections and code for reproducing results are publicly available at https://github.com/midas-network/covid19-scenario-modeling-hub https://doi.org/10.5281/zenodo.6584489.

### Role of the funding source

The funders had no role in study design, data collection and analysis, decision to publish, or preparation of the manuscript.

## Results

### Overall trajectory

The ensemble projected a declining COVID-19 incidence from September through December 2021 (Fig. 1). While national projections from different models largely agreed on trends and aligned with a decline in national case data through October 2021, substantial quantitative uncertainty remained (Fig. S1). The median national projections did not follow the trend of the rise in case counts and hospitalizations that started in November 2021, although as of early December 2021, observed trends largely were consistent with the uncertainty bounds.

**Fig. 2:**
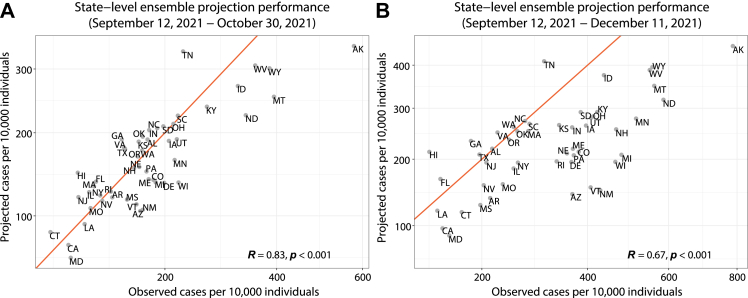
State-level ensemble projection performance from projection start date (September 12, 2021) through the observed weekly data available: A) before the assumed start of vaccination in 5–11 years old children (ending October 30, 2021; 6-week projection horizon) and B) after three months (ending December 11, 2021; 13-week horizon). Scenario A (childhood vaccination and no variant) projections are displayed. Projected cumulative cases vs. observed cumulative cases for all ages by state and normalized by state population. The red line marks where projected cumulative cases are equal to the corresponding state observations.

State-level ensemble projections of cumulative cases for Scenario A (vaccination of children 5–11 years old without a new variant) over the period of September 12, 2021 to October 30, 2021 were well-correlated with reported cumulative cases (Pearson correlation coefficient *R* = 0.83, p < 0.001) (Fig. 2), although observed trends deviated from the model-projected decline in November 2021 (projection Pearson correlation coefficient 13 weeks into projection period *R* = 0.67, p < 0.001). In particular, state-level observations tended to exceed ensemble projections for cumulative cases and deaths over this period. Based on ensemble projections, scenarios that assumed the emergence of a variant 50% more transmissible than the Delta variant in November 2021 led to models projecting a slow and moderate rise in national cases and deaths into early 2022 (Fig. 1). In scenarios that did not assume the emergence of a new variant, models projected that national cases would drop to a level similar to that observed in June 2021, and projected deaths would drop to a weekly incidence of less than one per 100,000 individuals by March 12, 2022.

### Vaccine benefits

**Fig. 3:**
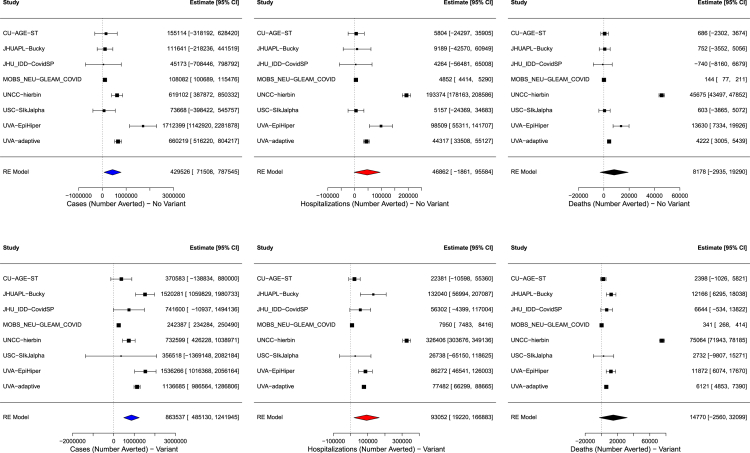
Scenario comparison to evaluate vaccine benefits. Absolute difference in estimates for all age cases, hospitalizations, and deaths when vaccination of 5–11-year-olds occurs without (top) and with (bottom) the emergence of a more transmissible variant from meta-analysis with random effects. Projection results from each team are analyzed as separate studies and are identified by team name abbreviation (see Supplemental Information for full team names). The size of each model-specific square is proportionate to the weight given to that model in the meta analysis. Corresponding cumulative values from the end of the projection period (March 12, 2022) for each model are provided in Supplemental Table S3.

**Fig. 4:**
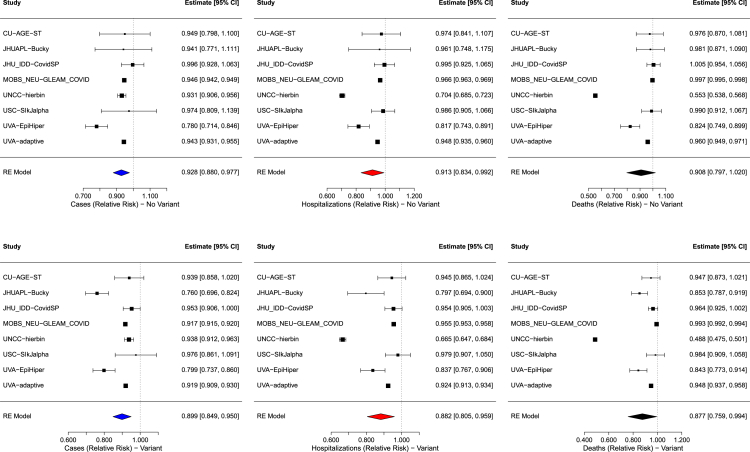
Scenario comparison to evaluate vaccine benefits. Incidence ratio estimates for all age cases, hospitalizations, and deaths when vaccination of 5–11 year-olds occurs without (top – scenarios A and B) and with (bottom – scenarios C and D) the emergence of a more transmissible variant from meta-analysis with random effects. See Table 1 for scenario definitions. The size of each model-specific square is proportionate to the weight given to that model in the meta analysis. Corresponding cumulative values from the end of the projection period (March 12, 2022) for each model are provided in Supplemental Table S3.

Assuming that vaccines showed strong effectiveness against the Delta variant and a hypothetical new variant, models projected that childhood vaccination would continue to reduce transmission burden across many important indicators (e.g., cases and hospitalizations). For the period November 1, 2021 to March 12, 2022, in the absence of a new variant, we estimated that vaccination of children 5–11 years old would avert ∼430,000 cases in the overall U.S. population (Fig. 3), a 7.2% reduction (mean incidence ratio [IR] 0.928 95% confidence interval [CI] 0.880–0.977, Fig. 4). With a more transmissible variant emerging in November 2021, the overall benefit of childhood vaccination increased to ∼860,000 cases averted, a reduction of 10.1% (mean IR 0.899, 95% CI 0.849–0.950). The models also projected that expanding vaccines to children could reduce overall population-level hospitalizations by 11.8% (mean IR 0.882, 95% CI 0.805–0.959) with the introduction of a new variant and by 8.7% (mean IR 0.913, 95% CI 0.834–0.992) without, corresponding to absolute reductions of ∼93,000 and ∼47,000 hospitalizations, respectively. Similarly, vaccination of children 5–11 years old was projected to reduce overall population-level deaths by 12.3% (mean IR 0.877, 95% CI 0.759–0.994) in the presence of a new variant and by 9.2% (mean IR 0.908, 95% CI 0.797–1.020) without a new variant (Fig. 3, Fig. 4).

At the state-level, the estimated effect of 5–11 year old vaccination varied substantially, with a median overall population-level estimated reduction in reported cases of 5.8% (median IR 0.942, IQR 0.918–0.950) in the scenarios without the emergence of a more transmissible variant (range of reductions across states: −7.5–22.0%). Projected case reductions for the whole population were more apparent at the state-level with the emergence of a highly transmissible variant, with a median reduction of 11.0% (reduction range −4.4–40.1%; median IR 0.890, IQR 0.818–0.914). Higher case reductions were projected to occur in states where vaccinated children 5–11 years old represent a higher proportion of the state population (Supplemental Fig. S2).

**Fig. 5:**
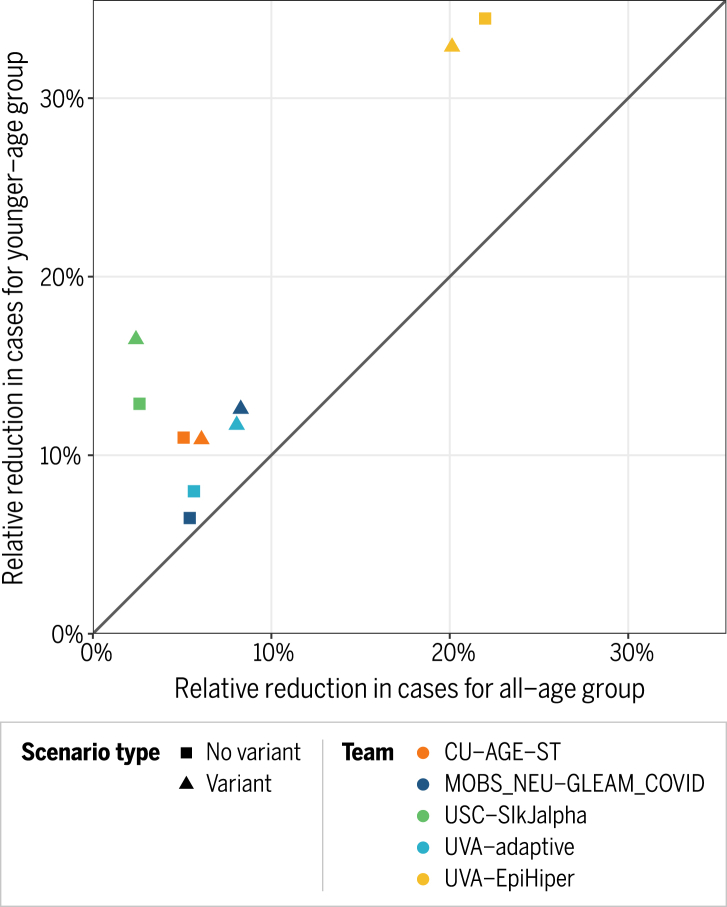
Comparison between younger-age projections and all-age projections for relative changes in cumulative cases where vaccination of 5–11-year-olds does not occur without (squares) and with (triangles) the emergence of a highly transmissible variant between November 1, 2021 and March 12, 2022. Younger-age groups considered are as follows: CU-AGE-ST (5–17 years), MOBS_NEU-GLEAM_COVID (0–11 years), USC-SlkJalpha (5–11 years), UVA-adaptive (0–17 years), UVA-EpiHiper (0–11 years).

Each of the five teams reporting younger age-group results projected that relative reductions in reported cases would be larger for the younger age group than for all ages (Fig. 5).

## Discussion

Immunization of children 5–11 years old likely provides both direct and indirect benefits and provides protection against potentially more transmissible SARS-CoV-2 variants. Our multi-model effort projected continued declines in cases, hospitalizations and deaths through March 2022, under the assumption that a highly transmissible new variant did not emerge, among other assumptions. Under the scenarios where a hypothetical variant was assumed to emerge in November 2021, immunization of children 5–11 years old resulted in larger relative reductions in cases, hospitalizations, and deaths among children than for the entire U.S. population, consistent with the existence of larger direct benefits to the age group vaccinated than (largely indirect benefits) overall.

Though immunizing 5-11 year-olds was projected by models to reduce cases by less than 10% overall (i.e., among all age groups), modest relative reductions translate to hundreds of thousands of cases, tens of thousands of hospitalizations, and thousands of deaths being averted. Population-level benefits are important because even though there is good direct protection afforded by the vaccine in most individuals, some groups (e.g., immunocompromised individuals, younger children, infants) rely on indirect protection.^28^

The new variant scenarios should be considered as a hypothetical stress test against increases in SARS-CoV-2 transmissibility that could highlight the benefits of vaccine expansion to a new age group. These scenarios should not be considered an illustration of what actually happened during the projection period September 2021–March 2022. SARS-CoV-2 transmission can increase for a variety of reasons. Here we investigated a hypothetical variant with 50% increased transmissibility over the Delta variant and without immune escape (or changes in severity). We did not model larger increases in transmissibility, although we would expect a consistent trend of a higher number of cases and deaths prevented by childhood vaccination as transmissibility increased.

There are clear differences between our hypothetical variant and the Omicron variant which rapidly emerged in South Africa in mid-November 2021.^29^^,^^30^ The Omicron variant has been shown to have a substantial degree of immune escape.^29^^,^^30^ Increased intrinsic transmissibility may also have contributed to its rapid spread,^31^ though this increase differs from that assumed in our variant scenarios. Prior work suggests that a moderate level of immune escape does not have substantial consequences unless paired with enhanced transmissibility, lending support to some transmission advantage.^32^ Further, differences in immunity acquired from natural infection or vaccination (and additionally by the type of vaccines and time since administration), the level of nonpharmaceutical interventions (NPIs), the prevalence of the Delta variant, and the severity of reinfections in different age groups, affect the trajectory and burden of the realized SARS-CoV-2 epidemic, and would in turn depart from our projections. In our later rounds of projections focused on Omicron (after vaccination was expanded to children 5–11 years old), we considered how uncertainties surrounding degrees of immune escape, transmissibility, and severity would affect epidemic trajectory and burden; however we did not explore vaccine benefits.^16^^,^^17^ Despite differences between Omicron and our hypothetical variant, the result that vaccinating children 5–11 years old provides some degree of population-level benefits in terms of reductions in cases, hospitalizations, and deaths, will likely hold. If the VE for symptomatic cases among children 5–11 years old was substantially lower than assumed by individual models in this set of projections, and/or if vaccine coverage among children was lower than hypothesized, the population level and direct benefits realized would likely be lower.

This work was undertaken to both inform policy concerning approval and recommendation of vaccines in 5–11 year-olds and to complement communication efforts. At the population level, vaccine benefits are determined by vaccine coverage and effectiveness, modulated by the susceptibility of and transmissibility from the 5–11 years old age group. To provide timely projections (e.g., for public release on September 22, 2021 at https://covid19scenariomodelinghub.org/),^16^ several assumptions were made, which we discuss in turn below.

Without known vaccine effectiveness in children at the time of projections, we assumed that vaccine effectiveness would be similar to that of adults. Assumptions for VE against symptomatic Delta infection differed between models, ranging between 60 and 95% after two doses of mRNA vaccines. Early data indicated a VE of 91% against symptomatic COVID-19 in 5–11 year olds,^33^ although updated estimates for 5-11 year-olds from the Omicron era are markedly lower (60.1%) and demonstrate marked waning after two months.^34^ Further, we assumed that immunization of children 5–11 years old would start on November 1, 2021; in fact the vaccine was approved on November 2 and some states expanded vaccine eligibility to 5-11 year-olds immediately following the approval. For vaccine coverage, we anticipated that more than 50% of U.S. children 5–11 years old would be vaccinated at the end of the projection period (defined by two doses received by March 12, 2022), guided by the uptake reported in adolescents during May–September 2021. However, this assumption was overly optimistic in that realized SARS-CoV-2 vaccine uptake was lower than the coverage levels assumed in our scenarios, reaching 26.3% at the national level and ranging across states from 9.4% to 57.2% (median: 23.5%, average: 26.1%).^2^

Our multi-model approach facilitates consideration of sources of epidemiological and situational uncertainty that affect projections of the direct and population-wide impacts of vaccination. We note that there is substantial variation in projected trajectories across the nine participating models (Fig. S1), particularly for scenarios that include the emergence of a novel variant. Multiple parameters and assumptions varied between models (Tables S1 and S2, Supplemental File S1) and we could not identify particular factors that drove differences in epidemic trajectory or vaccine benefits across models. Such variation is a benefit of our multi-model approach, with our ensemble projections synthesizing genuine scientific uncertainty. Areas of uncertainty include seasonality in transmission due to environmental conditions and behavior, waning of immunity, the expected levels of incidence in the coming months, and the potential role of children in transmission.

Age differences in susceptibility and transmissibility to COVID-19 is a persistent question that is particularly relevant for evaluation of childhood vaccination. Despite early reports of low symptomatic infections among children, there is now ample evidence that children get infected and transmit SARS-CoV-2, as shown by numerous school outbreaks.^35^^,^^36^ Serology studies indicate that 38% of children 5–11 years old had been infected by SARS-CoV-2 prior to November 2021, confirming that infection frequently occurs in this age group.^37^

There is empirical evidence that school closure and school-based mitigation measures reduce COVID-19 risk^38^; however reactive school closures and increased testing practices in schools were not considered by modeling groups. If vaccination induces immunity to SARS-CoV-2 infection in children 5–11 years old, then expanding vaccination to this age-group could reduce the potential need for prolonged school closures and other interventions directed towards this age-group. The indirect consequences of prolonged school closures are only beginning to be documented,^39^^,^^40^ but appear to be substantial and detrimental, particularly with respect to learning loss.^41^ Overall, there was agreement between models in projecting higher vaccine benefits in periods of high incidence, as illustrated by the new variant scenarios. Accordingly, by substantially reducing the risk of transmission in schools, childhood vaccination could reduce the need for closures or other controversial mitigation measures during epidemic surge.

Our study has several limitations. Only five modeling groups were able to provide age-resolved projections and the exact age groups differed between models due to different model structures and data inputs. Thus, it was not possible to estimate the direct effects of vaccination in children aged 5–11 years in this framework to compare with the results for the overall population. Additionally, our results only considered health outcomes (cases, hospitalizations, and deaths) and did not consider other important outcomes such as missed days of work or school, or the cost of medical care. Accounting for these considerations would tend to increase the benefits of vaccination.

Additionally, we projected the benefits of vaccinating 5-11 year-olds on a relatively short time scale of a few months. Longer-term benefits are difficult to estimate as they depend on the balance between duration of immunity in different age groups and viral evolution. As we move into the next stages of the pandemic, with immunity increasing in all age groups through natural infection and vaccination, a shift to endemic dynamics with annual wintertime outbreaks is expected.^42^ Incidence would also be projected to shift towards younger and immunologically naïve individuals.^43^^,^^44^ Based on observations of other pathogens,^45^^,^^46^ as the age distribution of disease becomes more concentrated in children, we expect direct and indirect vaccine benefits to increase in this age group.

Finally, the results in this paper are derived from scenario projections from a diverse set of individual models that are synthesized into a single set of projections. The accuracy of the conclusions rests on the validity of the counterfactual statements made by the ensemble projection. In the first weeks of projection (prior to the emergence of Omicron and the divergence of scenario assumptions), we find reasonable alignment between ensemble projections and observations at the state level (Fig. 2). However, it is challenging to retrospectively quantify scenario projection performance across the full 6-month time scale of the projections because the hypothesized scenarios will never be realized precisely as assumed. For example, while our analyses suggest the true vaccine impacts might be higher in the presence of a new more transmissible variant, the characteristics of the emergent Omicron variant vary from the specifics of our vaccination expansion scenarios, and thus should be interpreted with caution (see also^16^^,^^17^ for Omicron-specific projections). Additional research is needed to design approaches for performance evaluation of scenario projections. It will be important to resolve how to adjust for differences between scenario assumptions and the ultimately observed reality, and how to optimize approaches for multiple model aggregation in this particular context.

Continued occurrence of COVID-19 cases throughout 2022 in the U.S. and worldwide indicate that the pandemic is not ending. This collaborative modeling effort underscored the moderate direct and overall benefits of expanding the vaccination program to 5-11 year-olds across a range of different scenarios, including ones where the Delta variant remains dominant until mid-March 2022 and incidences gradually decline. Most importantly, increasing vaccination in children also builds resilience to potential increases in the upcoming pandemic trajectory, which may be fueled by new variants, waning immunity, increased contacts, or other unpredictable factors. The approach described here continues to be applied to increase the use of modeling for informing public health policy and communication. We hope that this work may also provide insight for future endeavors between modelers and policy makers in novel settings.

### Disclaimers

Any use of trade, firm, or product names is for descriptive purposes only and does not imply endorsement by the U.S. Government. The findings and conclusions in this report are those of the authors and do not necessarily represent the views of the National Institutes of General Medical Sciences, the National Institutes of Health, or the Centers for Disease Control and Prevention.

## Contributors

Rebecca K. Borchering, Luke C. Mullany, Emily Howerton, Claire P. Smith, Michelle Qin, Nicholas G. Reich, Harry Hochheiser, Jessica M. Healy, Rachel B. Slayton, Michael A. Johansson, Matthew Biggerstaff, Katriona Shea, Shaun A. Truelove, Michael C. Runge, Cécile Viboud, Justin Lessler contributed to conceptualization. Rebecca K. Borchering, Luke C. Mullany, Emily Howerton, Matteo Chinazzi, Claire P. Smith, Michelle Qin, Lucie Contamin, John Levander, Jessica Kerr, J Espino, Harry Hochheiser, Kaitlin Lovett, Matt Kinsey, Kate Tallaksen, Shelby Wilson, Lauren Shin, Joseph C. Lemaitre, Juan Dent Hulse, Joshua Kaminsky, Elizabeth C. Lee, Alison L. Hill, Jessica T. Davis, Kunpeng Mu, Xinyue Xiong, Ana Pastore y Piontti, Alessandro Vespignani, Ajitesh Srivastava, Przemyslaw Porebski, Srini Venkatramanan, Aniruddha Adiga, Bryan Lewis, Brian Klahn, Joseph Outten, Benjamin Hurt, Jiangzhuo Chen, Henning Mortveit, Amanda Wilson, Madhav Marathe, Stefan Hoops, Parantapa Bhattacharya, Dustin Machi, Shi Chen, Rajib Paul, Daniel Janies, Jean-Claude Thill, Marta Galanti, Teresa Yamana, Sen Pei, Jeffrey Shaman, Guido Espana, Sean Cavany, Sean Moore, Alex Perkins, Shaun A. Truelove, Michael C. Runge, Cécile Viboud, Justin Lessler contributed to data curation. Rebecca K. Borchering, Luke C. Mullany, Emily Howerton, Claire P. Smith, Michelle Qin, Cécile Viboud, Justin Lessler performed formal analysis. Luke C. Mullany, Emily Howerton, Lucie Contamin, John Levander, Jessica Kerr, J Espino contributed to software. Rebecca K. Borchering, Luke C. Mullany, Emily Howerton, Claire P. Smith, Michelle Qin, Lucie Contamin, John Levander, Jessica Kerr, J Espino, Justin Lessler contributed to visualization. Rebecca K. Borchering, Luke C. Mullany, Cécile Viboud, Justin Lessler wrote the original draft. All authors contributed to reviewing and editing drafts of the manuscript.

## Data sharing statement

Data and code for reproducing results are publicly available at https://github.com/midas-network/covid19-scenario-modeling-hub DOI: 10.5281/zenodo.6584489.

## Ethics committee approval

Not required.

## Declaration of interests

JL has served as an expert witness on cases where the likely length of the pandemic was of issue. MCR reports stock ownership in Becton Dickinson & Co., which manufactures medical equipment used in COVID-19 testing, vaccination, and treatment. JS and Columbia University disclose partial ownership of SK Analytics. JS discloses consulting for BNI. There are no other competing interests to declare.

## Appendix A Supplementary data

Supplementary Figs. S1–S3, Tables S1–S3 and Supplementary Method s
