# Supplementary material for: Impact of SARS-CoV-2 vaccination of children ages 5–11 years on COVID-19 disease burden and resilience to new variants in the United States, November 2021–March 2022: a multi-model study
Source: Lancet Reg Health Am. 2022 Nov 22;17:100398. doi: 10.1016/j.lana.2022.100398 (PMC9679449; doi:10.1016/j.lana.2022.100398)
Supplement: Supplementary Figs. S1–S3, Tables S1–S3 and Supplementary Method — s [file mmc1.docx]

**Impact of SARS-CoV-2 vaccination of children ages 5-11 years on COVID-19 disease burden and resilience to new variants in the United States, November 2021-March 2022: a multi-model study**

**Supplemental Methods:**

**COVID-19 Scenario Modeling Hub Round 9 Participating Teams:**

- Columbia University (New York, NY) – Age-Stratified Model (CU-AGE-ST)
- Johns Hopkins University (Baltimore, MD) ID Dynamics COVID-19 Working Group (JHU_IDD-CovidSP)
- Johns Hopkins University Applied Physics Lab (Laurel, MD) – Bucky (JHUAPL-Bucky)
- Northeastern University (Boston, MA) MOBS Lab – GLEAM COVID (MOBS_NEU-GLEAM-COVID)
- University of North Carolina at Charlotte (Charlotte, NC) – hierbin (UNCC-hierbin)
- University of Notre Dame (Notre Dame, IN) – FRED (NotreDame-FRED)
- University of Southern California (Los Angeles, CA) – SlkJalpha (USC-SlkJalpha)
- University of Virginia (Charlottesville, VA) – adaptive (UVA-adaptive)
- University of Virginia (Charlottesville, VA) – EpiHiper (UVA-EpiHiper)

**Projections**

Probabilistic projections were collected using 23 quantiles (i.e., 0.01, 0.025, 0.05, every 5% to 0.95, 0.975, and 0.99).

Five teams did not include waning (CU-AGE-ST, JHUAPL-Bucky, JHU_IDD-CovidSP, UNCC-hierbin, NotreDame-FRED, see above list of participating teams for full team names). Three teams assumed that immunity would wane exponentially with an average period of either six months (UVA-EpiHiper) or one year (MOBS_NEU-GLEAM-COVID, UVA-adaptive). The USC-SlkJalpha team assumed a weighted average of assumptions about waning.

Five teams also submitted projections with the same quantile structure for a younger age-group (0-11, 5-11, 5-17, and 0-17 year groups) and an older age-group when possible:

- CU-AGE-ST
- MOBS_NEU-GLEAM_COVID
- USC-SlkJalpha
- UVA-adaptive
- UVA-EpiHiper

For scenarios without the emergence of a more transmissible variant, mean reported case reductions in younger age-groups ranged from 6.5 to 34.5% across models over the November 1, 2021 to March 12, 2022 time period. Reductions in hospitalizations and deaths ranged from 5.6 to 34.5% and 5.2 to 34.5%, respectively. For scenarios with the emergence of a more transmissible variant, mean projected reductions in cases were 10.9 to 32.9%, with hospitalization and death reductions ranging from 9.0 to 32.9% and 5.9 to 33.1% respectively. Percent reductions were not estimated for one team, which reported zero deaths for the younger age-group over the November 1, 2021 to March 12, 2022 time period.

**Meta analyses**

To summarize the projected benefits of the vaccine program expansion across all modeling teams, we used a standard meta-analytic approach with random effects.^26^ We estimated the mean difference in cumulative incidence and the mean incidence ratio between scenarios with and without children 5-11 years old vaccinated, stratified by presence or absence of the new variant, for the portion of the projection period following the assumed start date of childhood vaccination (i.e., from November 1, 2021).

Specifically, for each scenario $s = A, B, C, D$, the modeling teams $m = 1,2,3 \ldots9$ directly provided us with the mean and variance (over their individual model replicates) of each cumulative outcome $o = Cases, Hospitalizations, Deaths$, at the start of the vaccination period ($t_{0}$) and the end of the of projection period ($t_{1}$) at the national level. We then estimated the mean ($\mu$) and variance ($\sigma^{2}$) for each model, scenario, and cumulative incidences over the period of interest as $\mu_{mso}= \mu_{mso}(-) - \mu_{mso}(t_{0})$and $\sigma_{mso}^{2}= \sigma_{mso}^{2}(t_{1}) + \sigma_{mso}^{2}(t_{0})$, respectively. To compare scenarios, for example A (with vaccination of children 5-11 years old) and B (without vaccination of children 5-11 years old), we then estimated the mean of the difference as $\mu_{mAo} - \mu_{mBo}$ and the variance of the difference as $\sigma_{mAo}^{2} +\sigma_{mBo}^{2}$. Note that in the absence of time-linked individual model trajectories/replicates, covariance between estimates (say between time points or between scenarios) was not estimated, and thus we conservatively assumed it to be zero. This almost certainly leads to overestimates of variance (true covariance is highly unlikely to be negative in this situation), thus yielding higher estimates of uncertainty and broader confidence intervals. We also estimated the incidence ratio as the ratio of the above means (e.g. $\frac{\mu_{mAo}}{\mu_{mBo}}$, with the proportion reduction due to the vaccine estimated as 1-$\frac{\mu_{mAo}}{\mu_{mBo}}$ ); the variance of this ratio was obtained using the delta method.^27^ For both absolute difference and incidence ratio, we estimated the standard error (SE) as $\sqrt{\sigma_{*}^{2}/n}$ , where $\sigma_{*}^{2}$ is the variance as defined above, and $n$ is the number of replicates (simulations or sets of projected outcomes) for that model. Model specific means and standard errors were combined via random effects meta-analysis using restricted maximum likelihood (REML).

At the state level, individual modeling teams provided quantile distributions, as specified above, but did not provide us with model-specific estimates of mean and variance over their replicates at the two above time points of interest. Therefore, to evaluate vaccine benefits at the state level, we estimated the mean and variance from the 23 available points from each model-specific cumulative distribution function (CDF) at $t_{0}$ and $t_{1}$(again, for each outcome, scenario, and state). Specifically, to each CDF (n~11,000; 51 states x 9 models x 3 outcomes x 4 scenarios x 2 timepoints) we fit a penalized cubic spline Poisson regression model to estimate a continuous quantile function, from which we simulated 25,000 replicates. The mean and variance of these replicates were then estimated, and the above-described approaches were followed to obtain mean and variance for the absolute difference and incidence ratio between scenarios, and for combining these using random effects REML meta analyses. This procedure was also followed for approximating the direct effect of vaccine expansion (within the younger age group).

**Supplemental Figures/Tables:**

**Figure S1: Model-specific trajectories for cases, hospitalizations, and deaths in each of the four scenarios.** Differences between models were generally most apparent in the new variant scenarios. Medians and 50% projection intervals are shown as solid lines and shaded areas respectively. In each panel, the vertical line indicates the last date for which data were able to be used for model calibration. Surveillance data to the right of the vertical line were observed after scenario projections were submitted. Models are distinguished by color. USC-SlkJalpha projected a large peak in cases in early 2022 in the variant scenarios. This peak was followed by peaks in hospitalizations and deaths. NEU-MOBs projected less of an impact in variant scenarios, with deep troughs observed at the national-level in early 2022. UVA-EpiHiper projected sharp decreases at the end of December 2021 and resurgences in the post-holiday period, driven by assumptions about school closures. See Round 9 tab of website^16^ for 95% projection intervals and additional visualization functionality (e.g., enlarging regions of these panels, viewing single models).

**Figure S2: State-level estimates for number of vaccinated 5-11 year olds per capita vs. projected cases occurring between November 1, 2021 and March 12, 2022.** Estimates for vaccinated 5-11 year olds are based on the vaccination coverage of 12-17 year olds on September 11, 2021 (the last day of data available for use in projections).


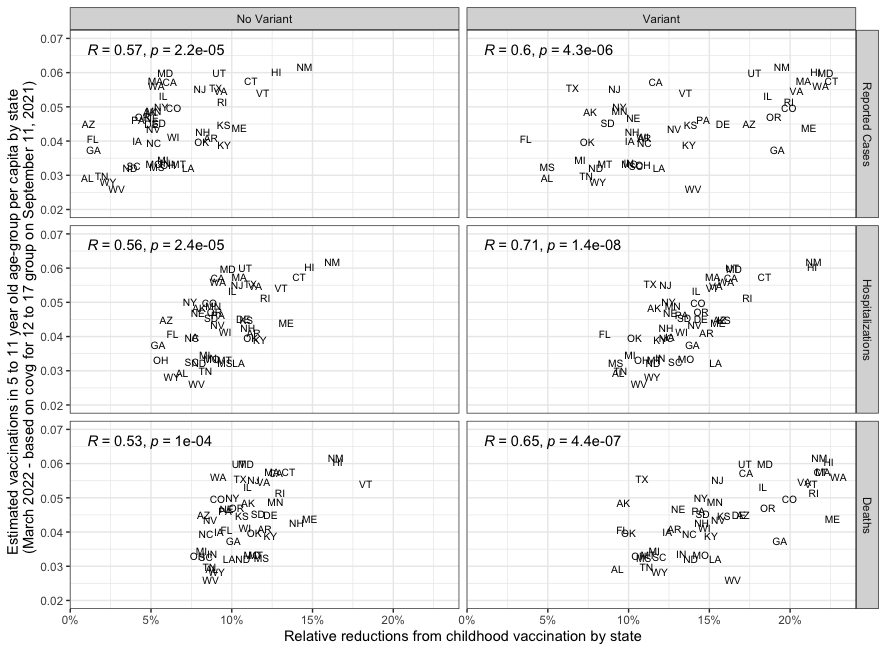


**Figure S3: Ensemble estimates of cumulative cases, hospitalizations, and deaths, by model for each of the four scenarios.** Ensemble median estimates are displayed as points and credible intervals of 50% and 95% are displayed as dark and light gray regions.


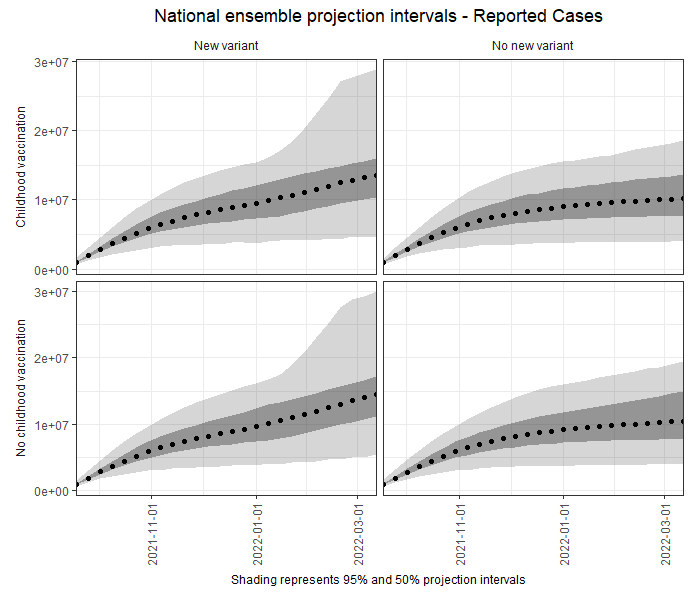

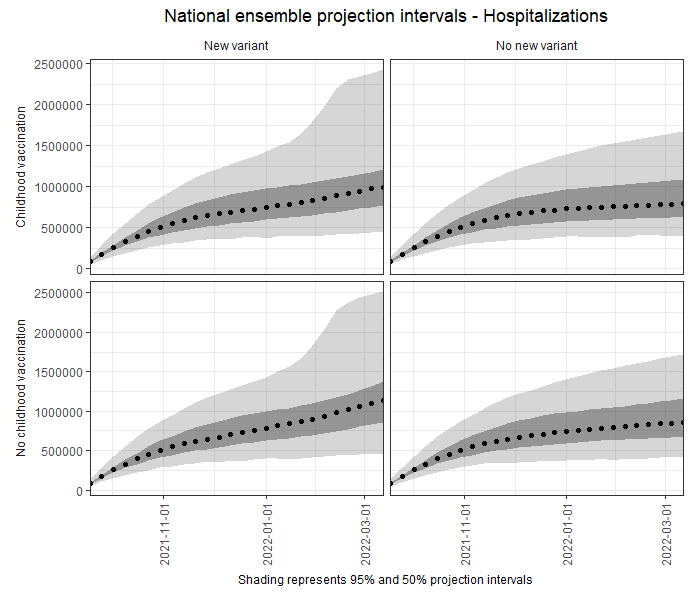

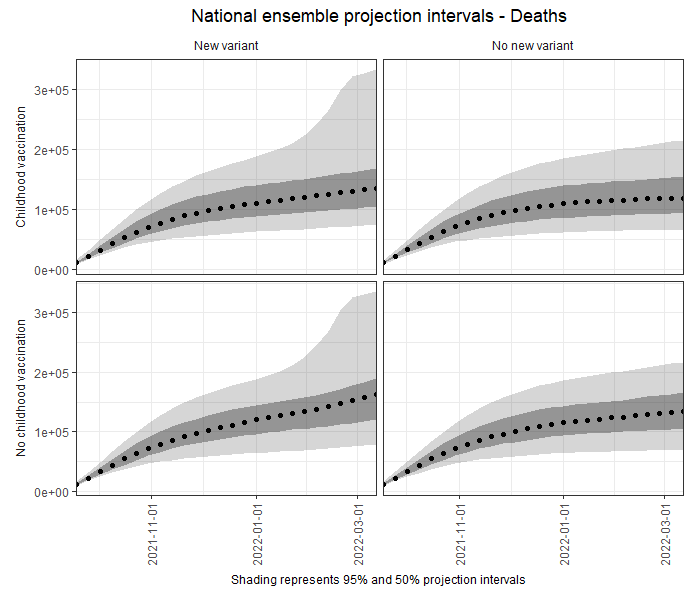


**Table S1: model-specific vaccine effectiveness (VE) assumptions for all age groups.** Since separate VE estimates for children were not available when scenarios were developed and projections were made, VE in children 5-11 years old was assumed to match those of older age groups.

| **Team** | **VE against infection** | **VE against symptoms** | **VE against hospitalization** |
| --- | --- | --- | --- |
| CU-AGE-ST | 30% 2 wks after 1st dose,  70% 2 wks after 2nd dose | 30% 2 wks 1st dose,  70% 2 wks after 2nd dose | 90% 2 wks after 2nd dose |
| JHU-APL-Bucky | means of 35% after 1st dose,  85% after 2nd dose  (drawn from a distribution) | same as VE against infection | same as VE against infection |
| JHU_IDD-CovidSP | 35% 2wks after 1st dose,  60% 2wks after second | 35% 2wks after 1st dose,  60% 2wks after second | 80% 2wks after second dose |
| NotreDame-FRED | non-Delta: 76%, Delta: 72% | non-Delta: 90%, Delta: 85% | non-Delta: 96%, Delta: 96% |
| MOBS-NEU-GLEAM_COVID | 32% 2wks after 1st dose,  60% 2wks after 2nd dose | 35% 2wks after 1st dose, 85% 2wks after 2nd dose | same as VE against symptoms |
| UNCC-Hierbin | 60%-85% for all age groups and virus variants | same as VE against infection | 90% for all age groups and virus variants |
| USC-SlkJalpha | 85% minus waning | 85% minus waning | (100% minus waning) if in immune state |
| UVA-Adaptive | same as VE against symptoms | 50% after after 1st dose,  95% after second,  67% after J&J dose,  minus waning | same as VE against symptoms |
| UVA-EpiHiper | same as VE against symptoms | 35% after after 1st dose,  85% after second,  60% after J&J dose | same as VE against symptoms |

**Table S2: additional model-specific assumptions including those about nonpharmaceutical interventions (NPIs).** Further model detail is included in the general model descriptions provided on the COVID-19 Scenario Modeling Hub website.^16^ Additional metadata corresponding to this round is available on the round 9 branch of the corresponding GitHub respository^17^ and in the additional Supplemental File: model metadata collected for Round 9 (external csv).

|  | **CU-AGE-ST** | **JHUAPL_Bucky** | **JHU_IDD-CovidSP** | **NotreDame-FRED** | **MOBS_NEU- GLEAM_COVID** | **UNCC-hierbin** | **USC-SIkJalpha** | **UVA-adaptive** | **UVA-EpiHiper** |
| --- | --- | --- | --- | --- | --- | --- | --- | --- | --- |
| **Model Type** | Compartmental | Meta-population compartmental | Meta-population compartmental | Agent-based | Meta-population compartmental | Trajectory tracking (non-mechanistic) | Discrete time heterogeneous rate compartmental | Meta-population compartmental | Agent-based |
| **Geography** | County | County | State | State | County | State | State | County | Individual-level |
| **Mobility and contact data** | SafeGraph mobility, POLYMOD contact rates | SafeGraph mobility, age-based contact matrices | Commuting | Google mobility | Google mobility, commuting, flight, age-based contact matrices | Not used | Cuebiq contact scores data to model future NPI changes | Not used | ACS Commute, National Household Travel Survey |
| **Age groups** | 5 | 16 | Age-adjusted for vacc, hosp, and death | Individual ages | 10 | None | 10 | Hosp and death outcomes adjusted for 3 age groups | 5 |
| **Waning of natural immunity** | None | None | None | None | None | None | None | None | Exponential with 6-month average time to partial susceptibility |
| **Waning of vaccine immunity** | None | None | None | None | Leaky vaccine | None | Leaky vaccine | None | Exponential with 6-month average time to partial susceptibility |
| **Importations** | No | No | No | Low constant importations per variant | Domestic/International | None | No | Low steady importation per county, 1 per million population | Low steady importation at state level |
| **Multiple strains** | Proportion of variant cases by time imported from simplified two-strain model | Proportion of variant cases estimated from simplified model | No | Co-circulating variants matching dominance data. | 2 strains | No | Separate model for each strain | No | Two strains |
| **NPI history** | Fitted transmissibility; no additional NPI relaxation | Fitted based on transmission rates | Fitted based on state-level reported periods of changing policy | Fitted based on historic deaths | Mobility + Oxford data | Based on historical data | Cuebiq data to assess current contact scores | Modeled by proxy using fitted transmissibility | Not modeled |
| **NPI relaxation** | Maximum relaxation reached | Linear | Linear | NPIs relaxed for the projection period | Linear | NPIs held constant | Linear | Linear | NPIs held constant |
| **R0 wild strain** | Estimates in each count based on observed cases | 2.4 (mean) | 2.3 | Transmission-specific parameters adjusted to data. R0 was not specified explicitly. | Prior in [1.6, 3.8]. Posterior defined by the model calibration in each state. | NA (due to non-mechanistic nature of the model) | Estimated from current data for current contact scores, and adjusted to pre-COVID contact scores | Variable, as we estimate intrinsic transmissibility and NPI effects through fitting of transmission rates from observed case series | NA (wild type not explicitly modeled, model initialized with delta variant with calibrated R_effective which varies among states) |

Note: additional assumptions that are left at the teams’ discretion are unspecified here to encapsulate current sources of scientific uncertainty (e.g., transmissibility assumptions).

**Table S3: End-of-projection period estimates (and credible intervals of 50% and 95%) of cumulative cases, hospitalizations, and deaths, by model for each of the four scenarios.** Note that the projection period ends March 12, 2022.

**Supplemental References**

**CU-AGE-ST**

Galanti M, Pei S, Yamana TK, Angulo FJ, Charos A, Khan F, et al. Non-pharmaceutical interventions and inoculation rate shape SARS-CoV-2 vaccination campaign success. Epidemiology and Infection. Cambridge University Press; 2021;149:e222.

<https://github.com/shaman-lab>

**JHUAPL_Bucky**

<https://github.com/mattkinsey/bucky>

**JHU_IDD-CovidSP**

Lemaitre JC, Grantz KH, Kaminsky J, Meredith HR, Truelove SA, Lauer SA, Keegan LT, Shah S, Wills J, Kaminsky K, Perez-Saez J. A scenario modeling pipeline for COVID-19 emergency planning. Scientific reports. 2021 Apr 6;11(1):1-3.

<https://github.com/HopkinsIDD/COVIDScenarioPipeline>

**NotreDame-FRED**

<https://github.com/confunguido/FRED>

**MOBS_NEU- GLEAM_COVID**

Chinazzi M, Davis JT, Ajelli M, Gioannini C, Litvinova M, Merler S, Pastore Y Piontti A, Mu K, Rossi L, Sun K, Viboud C, Xiong X, Yu H, Halloran ME, Longini IM Jr, Vespignani A. The effect of travel restrictions on the spread of the 2019 novel coronavirus (COVID-19) outbreak. Science. 2020 Apr 24;368(6489):395-400. doi: 10.1126/science.aba9757. Epub 2020 Mar 6. PMID: 32144116; PMCID: PMC7164386.

<http://covid19.gleamproject.org/>

**USC-SIkJalpha**

1. Srivastava A, Xu T, Prasanna VK. Fast and Accurate Forecasting of COVID-19 Deaths Using the SIkJalpha Model. arXiv preprint arXiv:2007.05180. 2020 Jul 10.

https://arxiv.org/abs/2007.05180

2. Srivastava A and Viktor K. Prasanna, Data-driven Identification of Number of Unreported Cases for COVID-19: Bounds and Limitations.SIGKDD Conference on Knowledge Discovery and Data Mining (KDD 2020) Health Day Track.

3. Srivastava A, Prasanna VK. Learning to Forecast and Forecasting to Learn from the COVID-19 Pandemic. arXiv preprint arXiv:2004.11372. 2020 Apr 23.

https://arxiv.org/abs/2004.11372

<https://scc-usc.github.io/ReCOVER-COVID-19>

**UVA-adaptive**

<https://biocomplexity.virginia.edu/project/covid-19-pandemic-response>

**Disclaimers**

Any use of trade, firm, or product names is for descriptive purposes only and does not imply endorsement by the U.S. Government.
