# Supplementary material for: Impact of SARS-CoV-2 vaccination of children ages 5–11 years on COVID-19 disease burden and resilience to new variants in the United States, November 2021–March 2022: a multi-model study
Source: Lancet Reg Health Am. 2022 Nov 22;17:100398. doi: 10.1016/j.lana.2022.100398 (PMC9679449; doi:10.1016/j.lana.2022.100398)
Supplement: Supplementary File S1 [file mmc2.docx]

Supplement 1: Supplemental methods, figures, tables, and references.

Supplemental Data File 1: Model metadata collected for Round 9 of the COVID-19 Scenario Modeling Hub (csv).
